# Complete Genome Sequence of the Novel Bacteriophage pSco-10 Infecting *Staphylococcus cohnii*

**DOI:** 10.1128/genomeA.01032-17

**Published:** 2017-11-22

**Authors:** Jin Woo Jun, Sib Sankar Giri, Hyoun Joong Kim, Cheng Chi, Saekil Yun, Sang Guen Kim, Sang Wha Kim, Jeong Woo Kang, Se Chang Park

**Affiliations:** Laboratory of Aquatic Biomedicine, College of Veterinary Medicine and Research Institute for Veterinary Science, Seoul National University, Seoul, Republic of Korea

## Abstract

Herein, we report the complete genome sequence of the *Staphylococcus Myoviridae* phage pSco-10 infecting *Staphylococcus cohnii*. The phage pSco-10 was isolated from duck feces collected from four farms in South Korea. The current report provides valuable information for genomic study of phages.

## GENOME ANNOUNCEMENT

Coagulase-negative staphylococci are recognized as important opportunistic pathogens since they have been frequently isolated from food animals and are related to antibiotic resistance (1). In our previous study, *Staphylococcus cohnii* SNUDS-2 was isolated from the brain tissue of ducks with tremors in South Korea (2). The antibiotic resistance patterns showed that SNUDS-2 was resistant to five antibiotics, cefoxitin, penicillin, oxacillin, ciprofloxacin, and sulfamethoxazole-trimethoprim. A virulent *Staphylococcus Myoviridae* phage infecting SNUDS-2, pSco-10, was isolated from duck feces collected from four farms in South Korea.

The phage’s genomic DNA was extracted using the SDS-proteinase K method as previously described (3) and sequenced by Macrogen in South Korea using standard shotgun sequencing reagents and a 454 GS-FLX Titanium sequencing system (Roche). Contig gaps were filled by additional PCR and primer walking. The genome contains no information for the physical ends of the phage. Potential open reading frames (ORFs) were predicted using GLIMMER and GeneMarkS (4, 5). The putative functions of the ORFs were analyzed by BLASTP searches at the National Center for Biotechnology Information (NCBI).

The genome size of pSco-10 is 101,986 bp, with a 31.11% total G+C content. A total of 132 ORFs were predicted in the genome, and 61 ORFs were determined to be functional. Sixty-one ORFs of pSco-10 were categorized into 5 functional groups as follows: (i) the phage structure and packaging proteins, 32 ORFs; (ii) the DNA replication and modification proteins, 18 ORFs; (iii) the signal transduction and regulatory protein, 1 ORF; (iv) the nucleotide metabolism proteins, 6 ORFs; and (v) the host lysis proteins, 4 ORFs. The comparative genome analysis of pSco-10 with *Staphylococcus* phage GH15 (GenBank accession number NC_019448) revealed that pSco-10 had 92% nucleotide sequence identity with GH15 and 79 homologous ORFs (59.84%) among its 132 ORFs. Although the sequence of pSco-10 showed high similarity to that of GH15, there are significant differences between the sequences of the two phages. pSco-10 has smaller genome size than that of GH15 (139,806 bp), which is the largest staphylococcal phage sequenced until now (6). pSco-10 contains several introns in four other genes in contrast to GH15, which did not contain any introns in its genome (6). No ORFs were found to be related to pathogenic factors.

In conclusion, the complete genome sequence of *Staphylococcus* phage pSco-10 will help to advance our understanding of the biodiversity of *Staphylococcus* phages. Also, the current study will provide valuable information for further genomic study of phages.

### Accession number(s).

The genome sequence of *Staphylococcus* phage pSco-10 was deposited in GenBank under the accession number KX011028.
